# Modified *Taq* DNA Polymerase for Allele-Specific Ultra-Sensitive Detection of Genetic Variants

**DOI:** 10.1016/j.jmoldx.2022.08.002

**Published:** 2022-11

**Authors:** Youngshin Lim, Il-Hyun Park, Huy-Ho Lee, Kyuwon Baek, Byung-Chul Lee, Ginam Cho

**Affiliations:** ∗ Department of Pathology, Brigham and Women’s Hospital, Harvard Medical School, Boston, Massachusetts; † GENECAST, Seoul, Republic of Korea

## Abstract

Allele-specific PCR (AS-PCR) has been used as a simple, cost-effective method for genotyping and gene mapping in research and clinical settings. AS-PCR permits the detection of single nucleotide variants and insertion or deletion variants owing to the selective extension of a perfectly matched primer (to the template DNA) over a mismatched primer. Thus, the mismatch discrimination power of the DNA polymerase is critical. Unfortunately, currently available polymerases often amplify some mismatched primer–template complexes as well as matched ones, obscuring AS detection. To increase mismatch discrimination, mutations were generated in the *Thermus aquaticus* (*Taq*) DNA polymerase, the most efficient variant was selected, and its performance evaluated in single nucleotide polymorphism and cancer mutation genotyping. In addition, the primer design and reaction buffer conditions were optimized for AS amplification. Our highly selective AS-PCR, which is based on an allele-discriminating priming system that leverages a *Taq* DNA polymerase variant with optimized primers and reaction buffer, can detect mutations with a mutant allele frequency as low as 0.01% in genomic DNA and 0.0001% in plasmid DNA. This method serves as a simple, fast, cost-effective, and ultra-sensitive way to detect single nucleotide variants and insertion or deletion mutations with low abundance.

Allele-specific PCR (AS-PCR), also known as allele-discriminating PCR, is commonly used to detect natural or experimentally introduced genetic variations.^1^^,^^2^ Originally developed in 1989 as an amplification-refractory mutation system,^2^ the premise of AS-PCR is a lower amplification efficiency of a mismatched primer–template complex compared with that of a matched one. Specifically, a more efficient (selective) extension of the primer by DNA polymerase occurs when the 3′ end of the primer is perfectly complemented (matched) to the template. For a matched primer–template complex, accurate Watson-Crick base pairing occurs at the 3′ end of the primer, while a mismatched primer–template complex results in non-canonical base pairing. If the selectivity of the DNA polymerase is high, only the primer with accurate base pairing to the template will be extended but not the primer with non-canonical base pairing, which is critical for accurate AS amplification.

Given its ease and simplicity, AS-PCR/allele-discriminating PCR has been used in many areas, including pharmacogenetics, genetic disorders, and cancer.^3^^,^^4^ For example, AS-PCR can be used to detect single nucleotide polymorphisms (SNPs) that can predict an individual’s susceptibility to disease and response to drugs^5^, ^6^, ^7^, ^8^ and can also trace the inheritance of specific genes and traits of interest.^9^^,^^10^ In addition to germline variants, detection of somatic variants has become increasingly valuable, notably in cancer as well as in hematologic, neurodevelopmental, and neurologic disorders.^11^, ^12^, ^13^, ^14^, ^15^ Furthermore, somatic variant detection in healthy individuals also provides important clues into normal development and aging of complex organs such as the brain.^16^, ^17^, ^18^ However, despite the power of this method in both clinical and research settings, the mismatch discrimination power of the currently available DNA polymerases is limited for making confident mutation calls.^19^

In addition to the selectivity, the sensitivity of the amplification is a critical factor for AS-PCR/allele-discriminating PCR, especially in early-stage disease detection. This is exemplified in the ability to discriminate and detect very small amounts of circulating tumor DNA (ctDNA) in blood, especially among the high abundance of other circulating cell-free DNA. The detection of ctDNA is particularly important for the early diagnosis of cancer, estimates of tumor volume, tracking recurrence, and monitoring therapy.^20^, ^21^, ^22^ Therefore, a rapid, sensitive, and accurate detection of somatic variants would enable better clinical care of patients with cancer.

To improve the specificity and sensitivity of AS-PCR/allele-discriminating PCR, *Thermus aquaticus* (*Taq*) DNA polymerase was altered by mutating amino acids that form close contacts with the phosphate backbone of the primer strand and identified a highly efficient triple mutant (TM)-*Taq* DNA polymerase. Using this engineered *Taq* DNA polymerase, the reaction buffer conditions as well as primer lengths were optimized. The efficiency of the improved *Taq* DNA polymerase was evaluated by using real-time quantitative PCR (qPCR) with SNP markers and cancer-related genes. Our results present a significantly improved polymerase and methodology that will enhance both investigative and clinical applications.

## Materials and Methods

### Mutagenesis of *Taq* DNA Polymerase Variants

**Figure 1:**
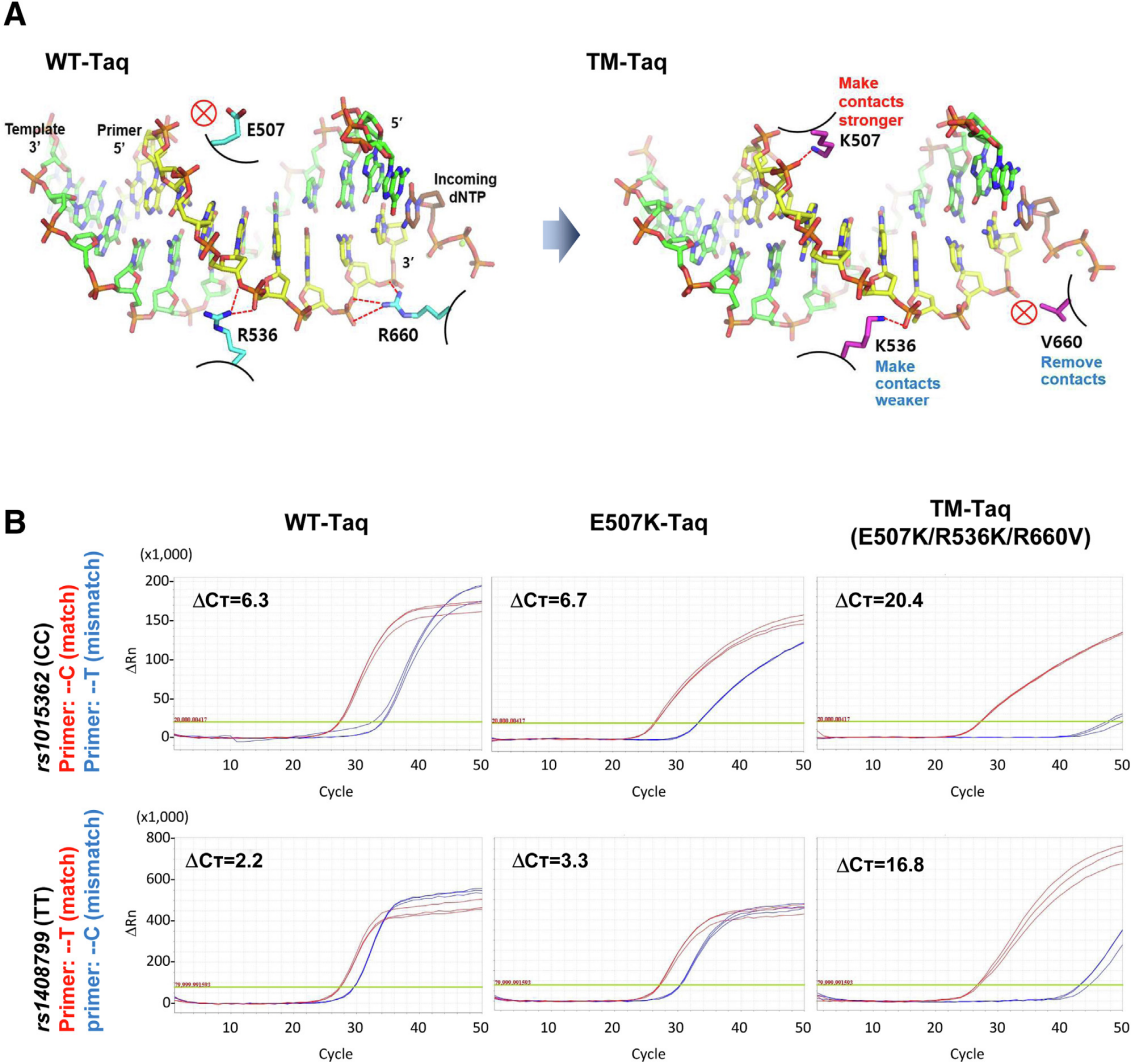
Engineering and single nucleotide polymorphism (SNP) genotyping test of *Taq* DNA polymerase for higher mismatch discrimination. **A:** Enzyme active site close-up illustrating the contacts between *Taq* DNA polymerase and primer–template DNA complex. Each indicated side chain of target residues of the wild-type (WT) (cyan) and mutant (purple) polymerases form different level of contacts with the backbone of the primer (yellow) bound to the template (green). **Dotted red lines:** hydrogen bonds between amino acid side chains of the enzyme and phosphate backbone of the primer; **circled red x:** no binding. **B:** Real-time quantitative PCR results for SNP detection by WT-, E507K-, and E507K/R536K/R660V-mutant *Taq* DNA polymerases. Template: genomic DNA (50 ng per reaction) from buccal swab. SNPs: *rs1015362* (CC genotype) and *rs1408799* (TT genotype). ΔC_T_: difference of C_T_ (cycle threshold; number of cycles required to reach 10% of maximum fluorescence) values between mismatched (blue) versus matched (red). ΔRn: difference of Rn (fluorescence signal of reporter probe normalized to that of reference dye) between the experimental versus baseline signal.

Mutant *Taq* DNA polymerase clones were generated by overlap extension PCR.^23^ First, intermediate PCR products (overlapping fragments of the entire product) were generated by using mutagenic primers and flanking primers; sequence information is presented in Table 1. The target PCR products were purified and used in a second PCR with Nco-F and Not-R primers to generate the full-length products. The amplified products were purified by gel electrophoresis and cloned into pET-28a(+) vector (Novagen, Gibbstown, NJ) using the EZ-Fusion HT Cloning Kit (Enzynomics, Daejeon, Republic of Korea). The ligation mixture was transformed into *Escherichia coli* DH5α, and the sequence was confirmed by Sanger sequencing. Wild-type (WT) and mutant *Taq* DNA polymerase structure modeling (Figure 1A) was conducted with the program PyMOL version 1.5 (Schrödinger, New York, NY) using the crystal structure of an active ternary complex of the *Taq* DNA polymerase I (Protein Data Bank code: 3KTQ).

**Table 1:** Oligonucleotide Sequences Used in This Study

Subset	Name	Sequences	Modification (5′/3′)
Mutagenesis	Nco-F	5′-AACTTTAAGAAGGAGATATACCATGCTGCCCCTCTTTGAGCC-3′	–
	E507K-R	5′-CTTGCCGGTCTTTTTCGTCTTGCCGAT-3′	–
	E507K-F	5′-ATCGGCAAGACGAAAAAGACCGGCAAG-3′	–
	R536K-R	5′-CTTGGTGAGCTCCTTGTACTGCAGGAT-3′	–
	R536K-F	5′-ATCCTGCAGTACAAGGAGCTCACCAAG-3′	–
	R660V-R	5′-GATGGTCTTGGCCGCCACGCGCATCAGGGG-3′	–
	R660V-F	5′-CCCCTGATGCGCGTGGCGGCCAAGACCATC-3′	–
	R536L-R	5′-CTTGGTGAGCTCCAGGTACTGCAGGAT-3′	–
	R536L-F	5′-ATCCTGCAGTACCTGGAGCTCACCAAG-3′	–
	Not-R	5′-TGGTGGTGCTCGAGTGCGGCCGCTCACTCCTTGGCGGAGAGCCAGT-3′	–
*rs1015362*	1015362 OF1	5′-TGAAGAGCAGGAAAGTTCTTCA-3′	–
	1015362 RC	5′-CTGTGTGTCTGAAACAGTG-3′	–
	1015362 RT	5′-CTGTGTGTCTGAAACAGTA-3′	–
	1015362 FAM	5′-TGCTGAACAAATAGTCCCGACCAG-3′	FAM/BHQ1
*rs1408799*	1408799 F2	5′-CCAGTGTTAGGTTATTTCTAACTTG-3′	–
	1408799 RT	5′-CTCGGAGCACATGGTCAA-3′	–
	1408799 RC	5′-CTCGGAGCACATGGTCAG-3′	–
	1408799 FAM	5′-AGATATTTGTAAGGTATTCTGGCCT-3′	FAM/BHQ1
*rs4911414*	4911414 R1	5′-AGTATCCAGGGTTAATGTGAAAG-3′	–
	4911414 FG	5′-GTAAGTCTTTGCTGAGAAATTCATTG-3′	–
	4911414 FT	5′-GTAAGTCTTTGCTGAGAAATTCATTT-3′	–
	4911414 FAM	5′-TGATGCTTTTCTCTAGTTGCCTTTAAGA-3′	FAM/BHQ1
*BRAF* V600E	V600E Fmt18	5′-TTTTGGTCTAGCTACAGA-3′	–
	V600E SR1	5′-GATCCAGACAACTGTTCAAACTG-3′	–
	V600E FAM	5′-AAATCTCGATGGAGTGGGTCCCATCA-3′	FAM/BHQ1
*JAK2* V617F	V617F Fmt22	5′-GTTTTAAATTATGGAGTATGTT-3′	–
	V617F OR1	5′-AGTCCTACAGTGTTTTCAGTTTC-3′	–
	V617F FAM	5′-TCAGTTTCAGGATCACAGCTAGGT-3′	FAM/BHQ1
*EGFR* L858R	L858R SF1	5′-CGTACTGGTGAAAACACCG-3′	–
	L858R Rmt14	5′-CAGCAGTTTGGCCC-3′	–
	L858R CFO560_R	5′-CAGCATGTCAAGATCACAGATTTTGGGC-3′	CFO560/BHQ1
	L858R Fmt19	5′-AGATCACAGATTTTGGGCG-3′	–
	L858R OR1	5′-TTGCCTCCTTCTGCATGGTATTC-3′	–
	L858R FAM_F	5′-CCAAACTGCTGGGTGCGGAAGAG-3′	FAM/BHQ1
*EGFR* Ex19Del	Ex19del SF2	5′-TCCTTCTCTCTCTGTCATAGGG-3′	–
	Ex19Del C1 Rmt19	5′-GTTGGCTTTCGGAGATGCC-3′	–
	Ex19Del CFO560_R	5′-CTCTGGATCCCAGAAGGTGAGAAAG-3′	CFO560/BHQ1
*EGFR* Ex20Ins	Ex20Ins SF4	5′-GAAGCCACACTGACGTGCC-3′	–
	Ex20Ins C3 Rmt18	5′-GGCACACGTGGGGGTTAC-3′	–
	Ex20Ins FAM_R	5′-TCACGTAGGCTTCCTGGAGGGA-3′	FAM/BHQ1
*GNAS* R844C	R844C FWT16	5′-GACCTGCTTCGCTGCC-3′	–
	R844C Fmt16	5′-GACCTGCTTCGCTGCT-3′	–
	R844X SR1	5′-GTTGACTTTGTCCACCTGGAA-3′	–
	R844X FAM_F	5′-CCTGACTTCTGGAATCTTTGAGACC-3′	FAM/BHQ1
*GNAS* R844S	R844S FWT16	5′-GACCTGCTTCGCTGCC-3′	–
	R844S Fmt16	5′-GACCTGCTTCGCTGCA-3′	–
	R844X SR1	5′-GTTGACTTTGTC-CAC-CTG-GAA-3′	–
	R844X FAM_F	5′-CCTGACTTCTGGAATCTTTGAGACC-3′	FAM/BHQ1
*GNAS* R844H	R844H FWT17	5′-GACCTGCTTCGCTGCCG-3′	–
	R844H Fmt17	5′-GACCTGCTTCGCTGCCA-3′	–
	R844X SR1	5′-GTTGACTTTGTCCACCTGGAA-3′	–
	R844X FAM_F	5′-CCTGACTTCTGGAATCTTTGAGACC-3′	FAM/BHQ1
*SMAD4* D351H	D351H RWT19	5′-AAGGGTCCACGTATCCATC-3′	–
	D351H Rmt19	5′-AAGGGTCCACGTATCCATG-3′	–
	D351H SF1	5′-AGGTAGGAGAGACATTTAAGGTTC-3′	–
	D351H FAM_R	5′-ACAGTAACAATAGGGCAGCTTGAAG-3′	FAM/BHQ1
*KIT* D816V	D816V FWT17	5′-TTTGGTCTAGCCAGAGA-3′	–
	D816V Fmt17	5′-TTTGGTCTAGCCAGAGT-3′	–
	D816V SR1	5′-CTTTGCAGGACTGTCAAGCA-3′	–
	D816V FAM_F	5′-AGGAAACGTGAGTACCCATTCTCTG-3′	FAM/BHQ1
*TP53* I195T	I195T RWT18	5′-AATTTCCTTCCACTCGGA-3′	–
	I195T Rmt18	5′-AATTTCCTTCCACTCGGG-3′	–
	I195-R196 SF1	5′-TGATTCCTCACTGATTGCTCT-3′	–
	I195-R196 FAM_R	5′-TAAGATGCTGAGGAGGGGCCAGA-3′	FAM/BHQ1
*PIK3CA* N345K	N345K RWT21	5′-TCAATGTCTCGAATATTTACA-3′	–
	N345K Rmt21	5′-TCAATGTCTCGAATATTTACC-3′	–
	N345K SF1	5′-GGGTTATAAATAGTGCACTCAGAATAA-3′	–
	N345K FAM_R	5′-AAATTCTTTGTGCAACCTACGTGAA-3′	FAM/BHQ1
*KRAS* G12V	G12V FWT17	5′-GTGGTAGTTGGAGCTGG-3′	–
	G12V Fmt17	5′-GTGGTAGTTGGAGCTGT-3′	–
	G12X SR5	5′-GTTGGATCATATTCGTCCACAAA-3′	–
	G12X FAM_F2	5′-AGGCAAGAGTGCCTTGACGATACA-3′	FAM/BHQ1
*KRAS* Q61R	Q61R RWT19	5′-ATTGCACTGTACTCCTCTT-3′	–
	Q61R Rmt19	5′-ATTGCACTGTACTCCTCTC-3′	–
	Q61R SF3	5′-AGTAGTAATTGATGGAGAAACCTG-3′	–
	Q61R FAM_R	5′-TGCTGTGTCGAGAATATCCAAGAGA-3′	FAM/BHQ1
*EGFR* C797S	C797S FWT21	5′-GCAGCTCATGCCCTTCGGCTG-3′	–
	C797S Fmt21	5′-GCAGCTCATGCCCTTCGGCTC-3′	–
	C797S SR2	5′-CAGGTACTGGGAGCCAATATTGTC-3′	–
	C797S FAM_F	5′-CTCCTGGACTATGTCCGGGAACAC-3′	FAM/BHQ1
*CDKN2A* L130Q	L130Q FWT16	5′-ATGTCGCACGGTACCT-3′	–
	L130Q Fmt16	5′-ATGTCGCACGGTACCA-3′	–
	L130Q SR1	5′-CCTTCCGCGGCATCTATG-3′	–
	L130Q FAM_F	5′-CACCAGAGGCAGTAACCATGCC-3′	FAM/BHQ1
*KRAS* K117N	K117N RWT18	5′-CTAGAAGGCAAATCACAT-3′	–
	K117N Rmt18	5′-CTAGAAGGCAAATCACAG-3′	–
	K117N SF1	5′-CCCAGAGAACAAATTAAAAGAGTTAA-3′	–
	K117N FAM_R	5′-ATTTCCTACTAGGACCATAGGTACATCTTC-3′	FAM/BHQ1
*KRAS* G13D	G13D SF1	5′-ATAAGGCCTGCTGAAAATGAC-3′	–
	G13D Rmt16	5′-GCACTCTTGCCTACGT-3′	–
	G13D Rmt15	5′-CACTCTTGCCTACGT-3′	–
	G13D Rmt14	5′-ACTCTTGCCTACGT-3′	–
	G13D FAM_R	5′-AGCTCCAACTACCACAAGTTTATATTCAGT-3′	FAM/BHQ1

### Expression and Purification of *Taq* DNA Polymerase Variants

Liquid culture of *E. coli* BL21(DE3) transformed with each recombinant DNA encoding *Taq* DNA polymerase variants and WT (200 mL, LB/kanamycin) was induced by 1 mmol/L of isopropyl-β-d-thiogalactopyranoside (at OD_600_: 0.4 to 0.5) for protein expression, and cells were harvested (after 12 hours) and resuspended in phosphate-buffered saline, 50 μL of which was removed for expression confirmation (SDS-PAGE, not shown). The remaining cells were harvested, resuspended in lysis buffer [20 mmol/L Tris-HCl (pH 8.0), 50 mmol/L potassium chloride (KCl), 1 mmol/L EDTA, 0.01% NP-40, 0.1% Tween-20, 1 mg/mL lysozyme, 1 mmol/L phenylmethylsulfonyl fluoride], sonicated, and incubated at 75°C for 1 hour with gentle shaking every 10 minutes at 4°C to remove the cell debris. After centrifugation, clarified lysates were loaded onto a low-pressure chromatography column (2 mL, Poly-Prep Columns; Bio-Rad Laboratories, Hercules, CA) packed with Q Sepharose XL (1 mL; Cytiva, Marlborough, MA). The column was then washed with 5 column volumes of the washing buffer [20 mmol/L Tris-HCl (pH 8.0), 50 mmol/L KCl, 1 mmol/L EDTA, 0.01% NP-40, 0.1% Tween-20], and the *Taq* DNA polymerase was eluted in a single step with 3 column volumes of the elution buffer [20 mmol/L Tris-HCl (pH 8.0), 500 mmol/L KCl, 1 mmol/L EDTA, 0.01% NP-40, 0.1% Tween-20].

The purified *Taq* DNA polymerase was diluted 10-fold with the dilution buffer [20 mmol/L Tris-HCl (pH 8.0), 1 mmol/L EDTA, 0.01% NP-40, 0.1% Tween-20] and loaded onto a second column, in the same manner described in the previous paragraph. The column was washed with 5 column volumes of the washing buffer, and the *Taq* DNA polymerase was eluted with 3 column volumes of the elution buffer. Dialysis (50 kDa; Spectra/Por 6 Dialysis Tubing, Spectrum Chemical, New Brunswick, NJ) was performed with a storage buffer [20 mmol/L Tris-HCl (pH 8.0), 100 mmol/L KCl, 0.1 mmol/L EDTA, 0.5% NP-40, 0.5% Tween-20, 50% glycerol] for stabilization of the eluted proteins. The E507K/R536K/R660V TM-*Taq* DNA polymerase is available through collaboration with the authors (GENECAST, Seoul, Republic of Korea).

### Primer and Probe Design

Each AS primer was designed according to its corresponding mutation or allele sequence. In the case of indels, two to four mismatches were designed at the 3′ end to distinguish the primer sequences according to the sequence difference before and after insertion or deletion. AS primers were designed with melting temperatures <60°C to increase selectivity due to mismatch at 3′ ends. To achieve sufficient binding efficiency, the primer designed on the opposite side of the AS primer was at, or slightly higher than, 60°C. Melting temperatures of oligonucleotides were calculated by using OligoAnalyzer 3.1 (Integrated DNA Technologies, Coralville, IA). Of note, in the TM-*Taq* PCR reaction, when the primer melting temperature was selected to be 2°C to 4°C lower than the annealing temperature, the polymerase reaction resulted in the greatest discrimination between the mutant and WT templates. The oligonucleotide sequences used in this study are listed in Table 1. Oligonucleotides were synthesized either by Integrated DNA Technologies (primers) or Biosearch Technologies (dual-labeled probes; Hoddesdon, UK).

### Template Preparation for qPCR

For SNP detection (*rs1015362*, *rs1408799*, and *rs4911414*), buccal swabs of volunteers (authors) were used to extract genomic DNA (Plain Dry Swab, Noble Bio, Hwaseong, Republic of Korea; QIAamp DNA Mini Kit, QIAGEN, Hilden, Germany). The genotype of each SNP was confirmed through Sanger sequencing. For cancer mutation detection with plasmid DNA, DNA fragments harboring each mutant and WT sequences were synthesized (Bio Basic, Markham, Ontario, Canada) and cloned into the pBluescript II SK(+) plasmid. Each template plasmid was linearized and diluted to 10^9^ copies/μL with TE buffer containing 2 ng/μL of sheared salmon sperm DNA (Invitrogen, Waltham, MA) through molecular weight calculation. The specific mutations used in this study included: *BRAF* V600E (c.1799T>A), *EGFR* C797S (c.2390G>C), *EGFR* L858R (c.2573T>G), *EGFR* Ex19Del (p.E746_T751delinsA, c.2237_2251del), *EGFR* Ex20Ins (p.D770_N771insG, c.2310_2311insGGT), *GNAS* R844C (c.2530C>T), *GNAS* R844H (c.2531G>A), *GNAS* R844S (c.2530C>A), *TP53* I195T (c.584T>C), *SMAD4* D351H (c.1051G>C), *KIT* D816V (c.2447A>T), *CDKN2A* L130Q (c.389T>A), *KRAS* G12V (c.35G>T), *KRAS* G13D (c.38G>A), *KRAS* Q61H (c.183A>C), *KRAS* K117N (c.351A>C), *PIK3CA* N345K (c.1035T>A), *PIK3CA* Q546L (c.1637A>T), and *PIK3CA* H1047R (c.3140A>G). For cancer mutation detection with genomic DNA, three human cell lines [HEK293T (ATCC, Manassas, VA), HEL 92.1.7 (ATCC), and A375SM (Korean Cell Line Bank, Seoul, Republic of Korea)] were grown in RPMI 1640 media supplemented with 10% fetal bovine serum (1% penicillin/streptomycin), and their genomic DNA samples were prepared by using the Blood & Cell Culture DNA Maxi Kit (QIAGEN). DNA concentrations were measured by using an Epoch microplate spectrophotometer (BioTek Instruments, Winooski, VT), and genomic DNA copy numbers were calculated by using the conversion factor of one copy of the haploid genome having a mass of 3.3 pg.

### qPCR Conditions

The reaction buffer contained 50 mmol/L Tris-HCl (pH 8.8), 75 mmol/L KCl, 5 mmol/L ammonium sulfate [(NH_4_)_2_SO_4_], 2.5 mmol/L magnesium chloride, 0.1% Tween 20, and 0.01% bovine serum albumin. Buffer optimization was achieved by starting with a buffer containing no monovalent ions [KCl, (NH_4_)_2_SO_4_] and variable amounts of KCl, (NH_4_)_2_SO_4_, or/and tetramethylammonium chloride with different combinations as indicated. qPCR was performed with 0.25 mmol/L of each dNTP, 200 nmol/L forward primer, 200 nmol/L reverse primer, 400 nmol/L dual-labeled fluorescent probe (Table 1), 2 μL of DNA template of desired copy number, and 15 ng of *Taq* DNA polymerase variants, in a total of 20 μL. The reactions were performed in a 7500 Fast Real-Time PCR System (Applied Biosystems, Waltham, MA) with an initial denaturation for 5 minutes at 95°C followed by the thermal cycles as given here: denaturation at 95°C for 10 seconds, annealing at 60°C for 30 seconds (fluorescence acquisition at this step), and elongation at 72°C for 10 seconds.

All qPCR data were analyzed in 7500 software (Applied Biosystems). In the qPCR reactions that were performed during earlier stages of the study (eg, Figure 1B), the reference dye carboxyrhodamine was not used, which resulted in higher ΔRn (difference of Rn values, where Rn is the fluorescence signal of the reporter probe normalized to that of the reference dye) values (more than thousand folds) than those in the reactions in which carboxyrhodamine was used. Of note, the presence or absence of carboxyrhodamine, however, is generally accepted to have no significant effects on qPCR reactions [ie, did not change threshold cycle (C_T_) values].

## Results

### Engineering *Taq* DNA Polymerase for Improved Mismatch Discrimination and Polymerization Activity

There have been previous efforts to improve the function of DNA polymerases by changing amino acids that affect the interaction between the polymerase and the primer–template complex.^19^^,^^24^, ^25^, ^26^ For example, substitution of the positively charged amino acids (R536, R587, or R660) of the *Taq* DNA polymerase Klenow fragment, which are directly in contact with the phosphate backbone of the primer in the closed conformation, has been shown to increase primer selectivity.^19^ However, when the single point mutations at these locations were combined to improve the selectivity, a drop in polymerase activity was observed.^19^ An E507K mutation reportedly stabilizes the *Taq* polymerase–DNA binary complex and improves polymerization speed, as it forms a strong interaction with a distal portion (location distant from the active site) of the primed template, dramatically reducing K_D_ (a dissociation constant).^27^^,^^28^ This mutation also enhances resistance to PCR inhibitors.^28^

To maximize mismatch discrimination and yet retain or improve polymerization activity of the *Taq* DNA polymerase, targeted mutagenesis was performed at amino acids E507, R536, and R660 to generate multiple variants in various combinations (Supplemental Table S1). Our model, based on X-ray crystallography structure of the active site of the enzyme, predicts that the substitution of the negatively charged amino acid (E, glutamate) at 507 to a positively charged one (K, lysine) would make a stronger contact with the primer; R536K (positive to positive; two hydrogen bonds to one) and R660V (positive to neutral; two hydrogen bonds to none) would generate a weaker and no contact, respectively (Figure 1A).^19^^,^^28^^,^^29^

### E507K/R536K/R660V TM-*Taq* DNA Polymerase Shows Excellent Selectivity in SNP Genotyping and Cancer DNA Mutation Detection

To evaluate the mismatch discrimination efficiency of the variant *Taq* DNA polymerases, they were first tested with a SNP genotyping assay. The performance of the WT- and mutant (E507 and E507K/R536K/R660V)-*Taq* DNA polymerases were compared in qPCR on two human SNPs (*rs1015362* and *rs1408799*) using genomic DNA from buccal swabs with two different sets of primers: matched and mismatched primers for each SNP (Figure 1B). The mismatch discrimination power of these enzymes was assessed by measuring ΔC_T_ values. ΔC_T_ is the difference in cycle threshold (C_T_, the number of cycles required for the fluorescence signal to exceed approximately 10% of the maximum fluorescence value) between the two reactions; for example, the reactions with matched versus mismatched primers (higher ΔC_T_ means higher discrimination) (Figure 1B). The single mutant *Taq* (E507K) showed a negligible level of increase in ΔC_T_ (6.7 and 3.3 for *rs1015362* and *rs1408799*, respectively) compared with the WT (6.3 and 2.2 for *rs1015362* and *rs1408799*). In contrast, the TM (E507K/R536K/R660V) enzyme showed a dramatic increase in ΔC_T_ (20.4 and 16.8 for *rs1015362* and *rs1408799*) compared with WT and single mutant, indicating that this variant enzyme amplifies DNA with the highest selectivity for the matched primer. The two double mutant *Taq* DNA polymerases, E507K/R536K and E507K/R660V, exhibited intermediate selectivity compared with WT (and the E507K single mutant) and the TM-*Taq* DNA polymerase (Supplemental Figure S1, A and B). Hereafter, the E507K/R536K/R660V TM-*Taq* DNA polymerase will be referred as TM-*Taq* DNA polymerase.

**Figure 2:**
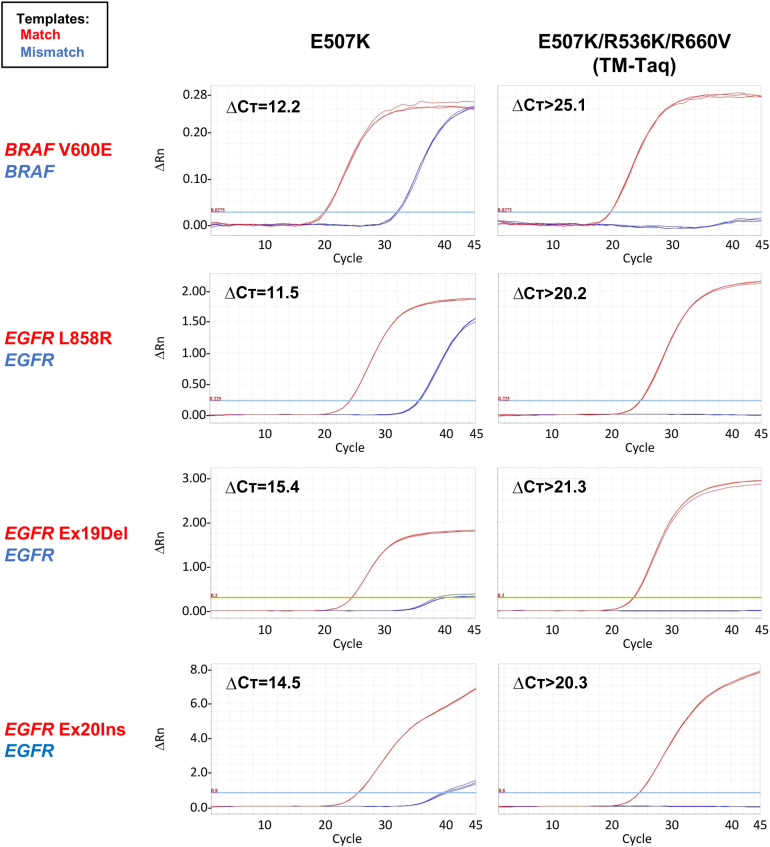
Triple mutant (TM)-*Taq* DNA polymerase shows excellent mismatch discrimination in cancer mutation detection. Real-time quantitative PCR results comparing the performance between E507K- and E507K/R536K/R660V-*Taq* polymerase. Templates: plasmid DNA harboring wild-type or mutant sequence of the *BRAF* and *EGFR* genes (1 × 10^6^ copies for *BRAF*, and 3 × 10^4^ copies for *EGFR* DNA) [*BRAF* V600E (c.1799T>A) and *EGFR* L858R (c.2573T>G), single nucleotide mutations; *EGFR* Ex19Del (c.2237_2251del), 15 bp in-frame deletion; *EGFR* Ex20Ins (c.2310_2311insGGT), 3 bp in-frame insertion]. Primers: each mutation-specific primer. ΔC_T_: the difference of C_T_ values between the wild-type (blue, mismatched) and mutant (red, matched) templates. ΔRn: difference of Rn (fluorescence signal of reporter probe normalized to that of reference dye) between the experimental versus baseline signal.

To further evaluate the mismatch discrimination of TM-*Taq* DNA polymerase, qPCR was performed with cancer genes. In these assays, two different cancer genes, *BRAF* and *EGFR,* were examined by using WT and cancer mutant plasmid DNA templates [single nucleotide (*BRAF* V600E and *EGFR* L858R) as well as deletion/insertion (*EGFR* Ex19Del and *EGFR* Ex20Ins) variants] and primers specific to cancer mutations (Figure 2). The performance of TM-*Taq* (E507K/R536K/R660V) was compared with that of a single mutant *Taq* DNA polymerase (E507K) (Figure 2). When E507K-*Taq* was used, it showed a modest discrimination between the matched versus mismatched primer–template complex (ΔC_T_ = 12.2, 11.5, 15.4, and 14.5 for four cancer mutations, respectively) (Figure 2). In contrast, when the TM-*Taq* was used, only the cancer mutant DNA templates (matched) were amplified, whereas the same number of copies (1,000,000 copies for *BRAF*; 30,000 copies for *EGFR*) of the WT templates (mismatched) were not detectable (ΔC_T_ >25.1, 20.2, 21.3, and 20.3 for four cancer mutations) (Figure 2). Together, these results show that the TM-*Taq* DNA polymerase displays highly selective amplification of the cancer allele over the WT allele, independent as to whether the cancer mutation is a single nucleotide variant or a deletion/insertion variant.

Of note, the R536K/R660V double mutant enzyme exhibited compromised polymerase activity even with the matched primer (only slow linear amplification without exponential amplification) (Supplemental Figure S2), as expected.^19^ In addition, the R536K single mutant enzyme showed a modest mismatch discrimination (ΔC_T_ = 10.5) for *EGFR* L858R detection, whereas the R536L mutant lost polymerase activity (Supplemental Figure S3). Another TM-*Taq* enzyme, E507K/R536L/R660V, with an R536L substitution instead of R536K had no polymerase activity (Supplemental Figure S3).

### TM-*Taq* DNA Polymerase Shows a Higher Mismatch Selectivity with all Possible Mismatch Types

The performance of the engineered TM-*Taq* enzyme was next tested with all 12 possible primer–template mismatch types (A/C, A/G, A/T, C/A, C/G, C/T, G/A, G/C, G/T, T/A, T/C, and T/G). The WT plasmid DNA templates for the selected cancer genes, and the two different primer sets for each gene, matched (WT primers) and mismatched (mutant primers), were used for qPCR runs with the WT-, E507K-, or TM-*Taq* DNA polymerases (Table 2). The C_T_ values were measured for each reaction, and the ΔC_T_ values (difference in C_T_ values between the matched versus mismatched) were compared among the WT-, E507K, and TM-*Taq* DNA polymerases in each mismatch case (Table 2).

**Table 2:** Performance Evaluation of the TM-*Taq* Enzyme with all 12 Possible Mismatch Types

Case	Type	Target mutation	Mutation at CDS (direction∗)	3′ end of WT primer (matched)	3′ end of MT primer (mismatched)	WT-*Taq* (C_T_)		E507K-*Taq* (C_T_)		TM-*Taq* (C_T_)		ΔC_T_ value		
						Matched	Mismatched	Matched	Mismatched	Matched	Mismatched	WT-*Taq*	E507K-*Taq*	TM-*Taq*
C/T	Transition	*GNAS* R844C	C>T (Forward)	C	T	26.2	27.5	26.2	27.8	26.2	38.4	1.3	1.6	12.2
C/A	Transversion	*GNAS* R844S	C>A (Forward)	C	A	26.2	39.0	26.2	40.6	26.2	49.7	12.8	14.4	23.5
C/G	Transversion	*SMAD4* D351H	G>C (Reverse)	C	G	26.7	36.3	26.7	36.7	26.4	41.9	9.6	10.0	15.5
A/G	Transition	*TP53* I195T	T>C (Reverse)	A	G	26.5	35.8	26.9	36.1	26.5	42.0	9.3	9.2	15.5
A/T	Transversion	*KIT* D816V	A>T (Forward)	A	T	25.8	37.7	26.3	38.1	25.8	>50†	11.9	11.8	>24.2‡
A/C	Transversion	*PIK3CA* N345K	T>G (Reverse)	A	C	28.6	36.2	28.9	37.0	27.7	>50†	7.6	8.1	>22.3‡
T/C	Transition	*KRAS* Q61R	A/G (Reverse)	T	C	26.3	31.5	26.3	32.3	25.4	42.0	5.2	6.0	16.6
T/G	Transversion	*KRAS* K117N	A/C (Reverse)	T	G	26.9	38.4	27.2	38.7	26.8	46.7	11.5	11.5	19.9
T/A	Transversion	*CDKN2A* L130Q	T>A (Forward)	T	A	27.6	39.0	27.7	40.0	27.1	>50†	11.4	12.3	>22.9‡
G/A	Transition	*GNAS* R844H	G/A (Forward)	G	A	26.3	31.0	26.3	32.4	26.0	40.6	4.7	6.1	14.6
G/T	Transversion	*KRAS* G12V	G/T (Forward)	G	T	27.0	32.2	27.0	33.0	26.6	46.4	5.2	6.0	19.8
G/C	Transversion	*EGFR* C797S	G/C (Forward)	G	C	27.6	40.4	27.6	42.0	27.1	>50†	12.8	14.4	>22.9‡

CDS, coding sequence; MT, mutant; *Taq*, *Taq* DNA polymerase; TM, triple mutant; WT, wild-type.

∗ Direction is defined as “forward” if the mutation case is taken from the coding strand and “reverse” if the mutation case is taken from the complementary strand.

† The mismatch product was not detected until the end of the cycles (50 cycles).

‡ C_T_ was assigned with the value of 50 as the highest cycle number assayed to calculate ΔC_T_: since the mismatch product was not detected until 50 cycles.

**Figure 3:**
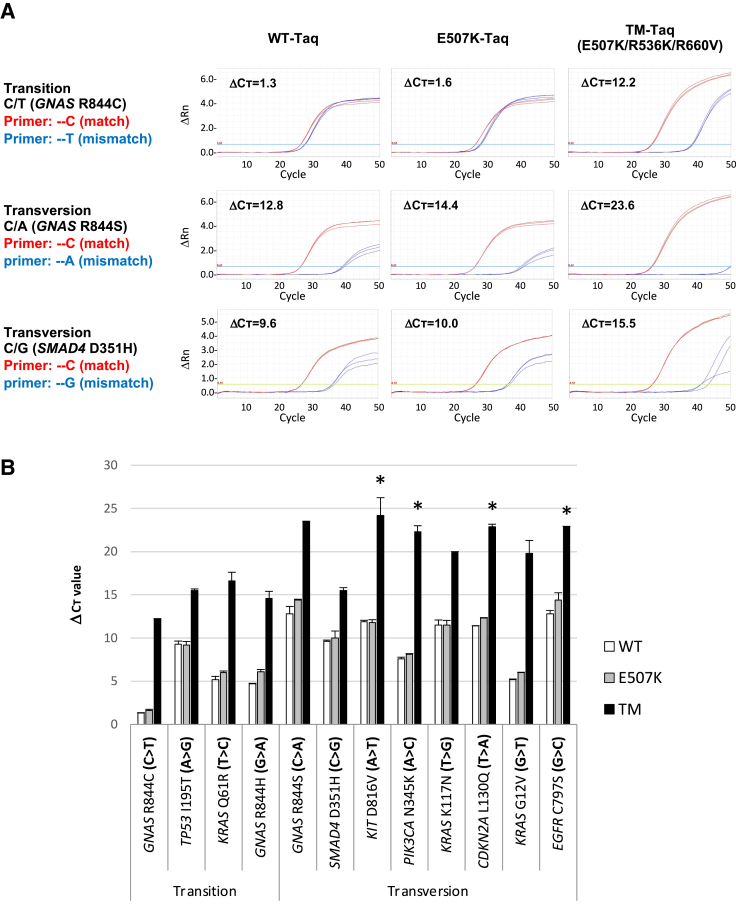
Triple mutant (TM)-*Taq* DNA polymerase shows improved mismatch selectivity for all 12 possible mismatch types. **A:** Real-time quantitative PCR results of the three mismatch types of the primer containing C at the 3′ end primer sequence (C to T, A, and G). Wild-type (WT) genomic DNA was used as a template with either WT-specific (red, match) or mutant-specific (blue, mismatch) primer in each reaction by WT-, E507K-, or TM-*Taq* DNA polymerase. **B:** Comparison of ΔC_T_ values (between the matched versus mismatched) among WT-, E507K-, and TM-*Taq* DNA polymerases for all 12 mismatch types. Details are listed in Table 2. The results of the other nine mismatch types are presented in Supplemental Figure S4. ΔC_T_: the difference of C_T_ values between the matched (red, WT) and mismatched (blue, mutant) template–primer complex. **Asterisks** indicate where ΔC_T_ values were calculated, not with the C_T_ obtained from the experiments, with the assigned C_T_ value of 50, because the PCR product from the mismatched primer–template complex was not detected until the end of the cycles (50 is the highest cycle number assayed).

The TM-*Taq* DNA polymerase showed improved mismatch discrimination in all possible mismatch cases, compared with the WT- or E507K-*Taq* DNA polymerase, although with differing degrees of allele discrimination depending on the mismatch type (Figure 3 and Supplemental Figure S4). For example, the TM-*Taq* DNA polymerase successfully distinguished C to T changes (pyrimidine to pyrimidine, a “transition” case), whereas WT- and E507K-*Taq* DNA polymerase did not (ΔC_T_ = 12.2 for TM-Taq, 1.3 for WT-*Taq*, and 1.6 for E507K-*Taq* DNA polymerase) (Figure 3A). Similarly, C to A and C to G changes (pyrimidine to purines, “transversion” cases) were also distinguished better by TM-*Taq* DNA polymerase (ΔC_T_ = 23.6) than by WT- or E507K-*Taq* (ΔC_T_ = 12.8 and 14.4, respectively), although WT- or E507K-*Taq* DNA polymerase also performed better with the transversion (ΔC_T_ = 12.8 and 14.4) than transition (ΔC_T_ = 1.3 and 1.6) cases (Figure 3A). Likewise, the other nine of the mismatch cases were all tested and produced similar results (Supplemental Figure S4 and Table 2). As expected, overall mismatch discrimination by all three enzymes was better in transversion cases than in transition cases (Figure 3B).

### Primer Design and Buffer Condition Can Affect the Mismatch Selectivity of TM-*Taq* DNA Polymerase

To optimize qPCR reaction for TM-*Taq* maximum mismatch discrimination,^30^ the effect of primer length was first evaluated on the selectivity of extension by TM-*Taq* DNA polymerase. For this, *KRAS* G13D mutant and WT plasmid DNAs were used as templates, with three different lengths of primers (14-, 15-, and 16-mers) specific (matched) to *KRAS* G13D (Supplemental Figure S5A). Although a C_T_ delay of about 2 was observed in the matched primer (compare C_T_ = 28 in 15- or 16-mer vs C_T_ = 30 in 14-mer), the 14-mer primer exhibited optimal discrimination. These data suggest that optimization of the primer length is critical for a successful mismatch discrimination in TM-*Taq* DNA polymerase–mediated AS-PCR. Similar results were observed for *PIK3CA* Q546L and H1047R mutant detection; 19-mer (*PIK3CA* Q546L) or 18-mer (*PIK3CA* H1047R) primers resulted in the most effective mismatch discrimination by TM-*Taq* enzyme (Supplemental Figure S5A).

Next, the ideal PCR reaction buffer conditions were determined. Starting concentrations were 50 mmol/L Tris-HCl (pH 8.8), 0.1% Tween 20, 0.01% bovine serum albumin, and 2.5 mmol/L magnesium chloride as a base and the concentration and combination of KCl, (NH_4_)_2_SO_4_, or tetramethylammonium chloride, which are known to affect the specificity and sensitivity of the reaction, were formulated.^31^ The titration curves indicate that 80 mmol/L KCl or 80 mmol/L tetramethylammonium chloride resulted in the optimal mismatch discrimination for *KRAS* Q61H and WT templates (Supplemental Figure S5B). Through a further optimization process with other template/primer pairs, it was found that the combination of 75 mmol/L KCl and 5 mmol/L (NH_4_)_2_SO_4_ worked the best overall (Supplemental Figure S5C) and thus was used for all the TM-*Taq* PCR reactions.

Taken together, the AS-PCR condition for TM-*Taq* DNA polymerase to detect rare mutations (eg, cancer-specific somatic mutations widely used as biomarkers) was optimized (primer/probe, buffer condition, and TM-*Taq* enzyme) and referred to as an allele-discriminating priming system (ADPS) (Supplemental Figure S6).

### Ultra-sensitive Detection of Rare Somatic Mutations by TM-*Taq* DNA Polymerase–Based ADPS

**Figure 4:**
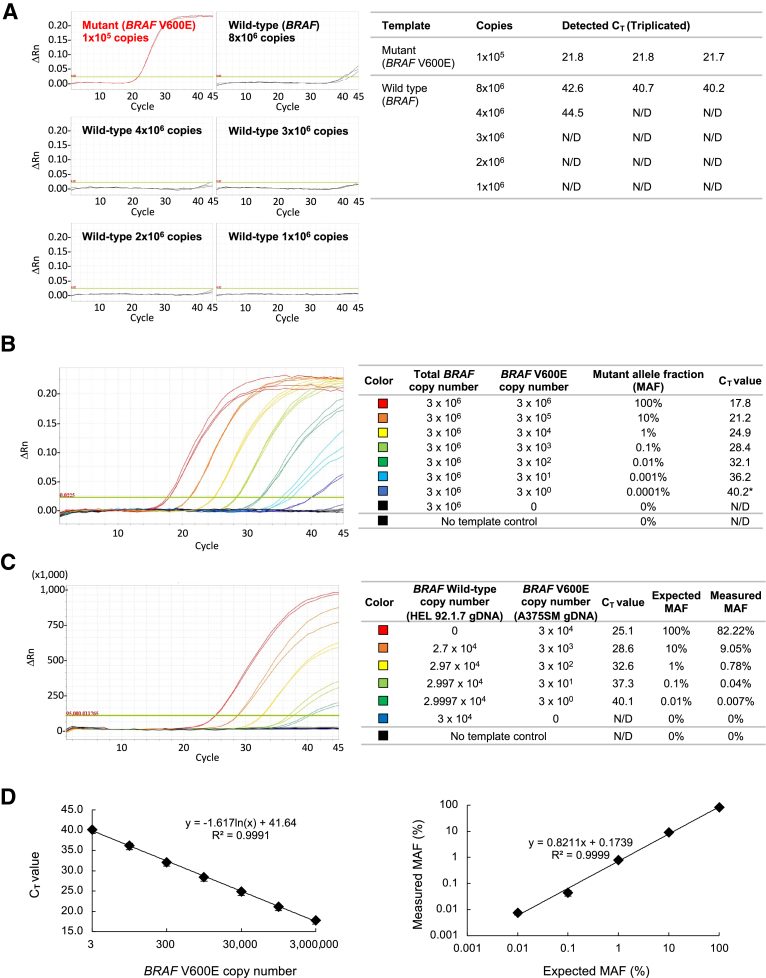
Ultra-sensitive detection of the rare mutations by the triple mutant-*Taq* DNA polymerase–based allele-discriminating priming system. **A:** Evaluation of specificity [real-time quantitative PCR (qPCR) results]. Templates: mutant (1 × 10^5^ copies) or increasing amount of wild-type (WT) (1 × 10^6^ to 8 × 10^6^ copies) plasmid DNA templates. **B:** Sensitivity test with plasmid DNA templates (qPCR results). Templates: plasmid blend samples (WT and mutant *BRAF*) with mutant allele fraction (MAF) ranging from 0.0001% to 50%. In the case of the 0.0001% sample, only two wells were detected out of the triplicated test (as indicated by the **asterisk**). **C:** Sensitivity test with genomic DNA (gDNA) templates (qPCR results). Templates: gDNA blend (WT and mutant *BRAF*) from HEL92.1.7 (*BRAF* WT) and A375SM (homozygous *BRAF* V600E), with MAF ranging from 0.01% to 100%. **D:** (**Left**) The mean C_T_ value plotted against the copy number (logarithm) of the mutant plasmid DNA template present in each sample from Figure 4B. Error bar: standard deviation. (**Right**) Measured MAF plotted against the expected MAF of the mutant template (gDNA). For measured MAF, copy number of the mutant template (*BRAF* V600E) (genomic DNA) was first calculated from the C_T_ value obtained in each reaction (using the linear regression equation in the standard curve on the left) and then converted to MAF. Primer: *BRAF* V600E-specific primer for **A**, **B**, and **C**. N/D, not detected.

To evaluate the sensitivity of the TM-*Taq* enzyme in ADPS, the maximum abundance of the DNA template for the mutant enzyme was first determined to appropriately return a negative result with a mismatched primer. For this, the DNA amplification was monitored in multiple reactions with different input copy numbers of the WT *BRAF* plasmid DNA template, ranging from 1 × 10^6^ to 8 × 10^6^, while the *BRAF* V600E-specific primer (mismatched to template) was used (Figure 4A). With up to 3 × 10^6^ copies of the WT template, no amplification was detected with a mutant-specific primer, although 4 × 10^6^ and 8 × 10^6^ copies resulted in a negligible level of amplification [one reaction of three with 4 × 10^6^ (C_T_ = 44.5) and all three reactions with 8 × 10^6^ (average C_T_ = 41.2)] (Figure 4A). Thus, the maximum input copy number of the WT template without generating false-positive amplicons is 3 × 10^6^. As a positive control, 1 × 10^5^ copies of the *BRAF* V600E mutant plasmid DNA template (matched) produced successful amplification (average C_T_ = 21.8) (Figure 4A). These results establish that no false-positive amplicons are detected, when up to 3 × 10^6^ copies of WT plasmid DNA are used as the only source of the template.

The sensitivity of TM-*Taq* enzyme was next assessed to detect *BRAF* V600E template when an excess amount of WT template was present in the same reaction. A serial dilution of the plasmid DNA template was prepared by adjusting the proportion of the cancer mutant DNA relative to the WT DNA, with the mutant (minor) allele fraction (MAF) ranging from 100% to 0.0001% (Figure 4B). When 3 × 10^6^ copies of the WT plasmids were present in the sample, the mutant template was amplified by TM-*Taq* DNA polymerase, down to 0.0001% MAF (Figure 4B). Reassuringly, the C_T_ value and the amount of the *BRAF* V600E template present in each reaction showed an inverse linear relationship (Figure 4D).

Furthermore, the results from plasmid DNA were confirmed with genomic DNA extracted from cells (Figure 4C). The genomic DNA from the HEL92.1.7 cell line in which the *BRAF* V600 region is WT and from the A375SM cell line containing the *BRAF* V600E mutation (homozygous) were mixed. Even in the presence of 3 × 10^4^ copies of the WT genomic DNA templates, as few as 0.01% of the mutant templates were successfully amplified, indicating a highly sensitive detection of the mutations with ultra-low MAF, by TM-*Taq* enzyme-mediated AS-PCR (Figure 4C).

**Figure 5:**
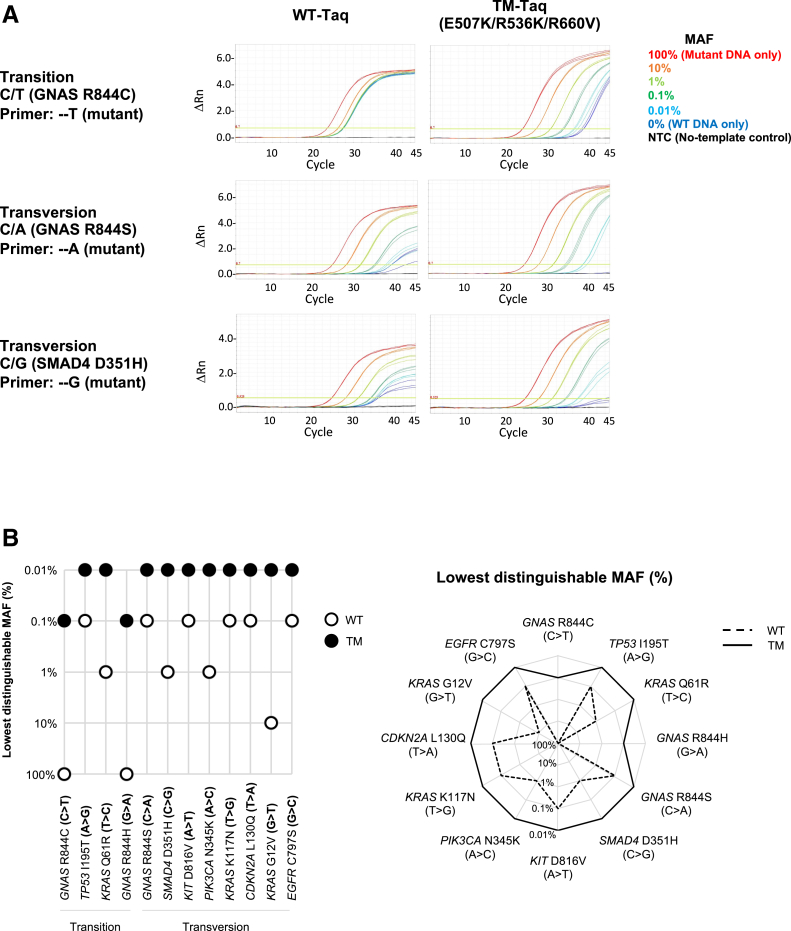
Comparison of the limit-of-detection (sensitivity) between the wild-type (WT)- and triple-mutant (TM)-*Taq* DNA polymerases with all possible mismatched types. **A:** Real-time quantitative PCR results of the three representative mismatch types (C to T, A, and G) using the selected cancer mutant DNA templates (plasmid DNA) mixed with WT templates (genomic DNA) at different proportion [mutant allele fraction (MAF) from 100% to 0%] and the mutant primers, by WT- or TM-*Taq* DNA polymerase. **B:** Two different plots summarizing the lowest distinguishable MAF of the WT- and TM-*Taq* DNA polymerases with all 12 possible mismatch types.

Finally, the limit of detection (sensitivity) between the WT- and TM-*Taq* DNA polymerases was compared with all 12 possible mismatch examples (Table 3). For this, qPCR was performed with either enzyme in the presence of: i) the selected cancer mutant plasmid DNA templates (Table 3) mixed with each WT genomic DNA (from HEK293T cells), with the MAF ranging from 100% (ie, mutant DNA only) to 10%, 1%, 0.1%, 0.01%, and 0% (ie, WT DNA only); and ii) the mutant primers. The results were compared for each MAF between each enzyme (Figure 5A and Supplemental Figure S7). Given that even the WT DNA templates (0% MAF) can be also amplified with the mutant primers if allowed with enough cycle number, the selective amplification was called successful only if the difference between the C_T_ value of the reaction with a certain MAF (X% MAF) and that with the 0% MAF (thus only WT DNA present) is bigger than 2 (ΔC_T_ >2; ΔC_T_ = C_T0% MAF_ – C_Tx% MAF_). Using this criterion, the lowest distinguishable MAF (%) was determined for each mismatch case in each enzyme (Figure 5B). The lowest distinguishable MAF for the TM-*Taq* DNA polymerase was 0.01% in 10 of 12 mismatch cases and 0.1% for the other two mismatch cases (C>T in *GNAS* R844C and G>A in *GNAS* R844H), suggesting consistently higher sensitivity, regardless of the mismatch types. On the contrary, WT-*Taq* DNA polymerase showed a lower and variable degree of sensitivity depending on the mismatch types, compared with the TM-*Taq* enzyme.

**Table 3:** Limit-of-Detection Comparison between WT- and TM-*Taq* with all Mismatch Cases

Case	Target	*Taq* DNA polymerase	C_T_ for each reaction with the indicated MAF					
			100%	10%	1%	0.10%	0.01%	0% (WT only)
C/T	*GNAS* R844C	WT-*Taq*	**23.5**	26.1	27.3	27.4	27.5	27.5
		TM-*Taq*	**23.6**	**27.0**	**30.5**	**33.6**	36.5	38.4
C/A	*GNAS* R844S	WT-*Taq*	**24.3**	**27.7**	**31.1**	**34.7**	37.9	39.8
		TM-*Taq*	**24.3**	**27.7**	**31.3**	**34.5**	**38.8**	N/D∗
C/G	*SMAD4* D351H	WT-*Taq*	**24.4**	**27.8**	**30.9**	**34.0**	**35.1**	35.7
		TM-*Taq*	**24.3**	**27.7**	**30.9**	**34.3**	**37.7**	43.5
A/G	*TP53* I195T	WT-*Taq*	**24.6**	**27.9**	**31.3**	**34.4**	36.5	36.9
		TM-*Taq*	**24.7**	**28.1**	**31.5**	**35.0**	**38.2**	43.2
A/T	*KIT* D816V	WT-*Taq*	**24.5**	**27.8**	**31.3**	**34.5**	38.4	38.8
		TM-*Taq*	**24.9**	**27.7**	**31.3**	**34.3**	**37.8**	N/D∗
A/C	*PIK3CA* N345K	WT-*Taq*	**25.6**	**28.9**	**32.6**	35.4	36.7	36.9
		TM-*Taq*	**25.6**	**29.0**	**32.4**	**35.7**	**39.2**	N/D∗
T/C	*KRAS* Q61R	WT-*Taq*	**24.7**	**27.9**	**31.1**	32.4	33.3	33.2
		TM-*Taq*	**24.7**	**28.2**	**31.8**	**35.0**	**37.9**	42.8
T/G	*KRAS* K117N	WT-*Taq*	**25.0**	**28.3**	**31.7**	**34.6**	37.5	37.6
		TM-*Taq*	**24.8**	**28.2**	**31.3**	**34.6**	**38.0**	43.0
T/A	*CDKN2A* L130Q	WT-*Taq*	**24.3**	**27.6**	**31.0**	**34.4**	37.3	38.9
		TM-*Taq*	**23.9**	**27.4**	**30.7**	**34.0**	**38.0**	N/D∗
G/A	*GNAS* R844H	WT-*Taq*	**25.5**	28.4	29.9	30.2	30.2	30.2
		TM-*Taq*	**25.4**	**28.9**	**32.5**	**36.2**	39.2	40.8
G/T	*KRAS* G12V	WT-*Taq*	**24.6**	**27.9**	30.9	32.3	32.7	32.7
		TM-*Taq*	**24.4**	**27.8**	**31.1**	**34.6**	**37.5**	42.2
G/C	*EGFR* C797S	WT-*Taq*	**25.2**	**28.6**	**32.0**	**35.4**	38.3	39.5
		TM-*Taq*	**24.6**	**28.0**	**31.5**	**35.1**	**38.2**	N/D∗

The bold numbers indicate where the enzyme was able to distinguish the mutant allele from the wild-type (WT) [we defined if ΔC_T_ >2 (ΔC_T_ = C_T0% MAF_ - C_Teach%MAF_), then distinguishable]. Note 0% mutant (or minor) allele fraction (MAF) indicates WT template only. TM, triple mutant.

∗ Not detected (N/D); ie, the fluorescent signal-PCR product is not detected when checked up to 50 cycles of reactions.

Taken together, these data show a highly selective and ultra-sensitive detection of rare somatic mutations in cancer genes using the TM-*Taq* enzyme-based ADPS established in this study.

## Discussion

Among the various methods used to detect and analyze SNPs/single nucleotide variants, primer–template complex mismatch discrimination-based qPCR is fast, simple, and cost-effective. Despite this benefit, the low selectivity of the polymerase has been a limiting factor. Here we report an engineered *Taq* DNA polymerase that exhibits high selectivity and sensitivity for single nucleotide mismatch detection.

The crystal structure of the *Taq* DNA polymerase^29^^,^^32^ has shown that the phosphate backbone of the nine nucleotides at the 5′ end of the DNA template interacts with the DNA polymerase. The seven nucleotides at the 3′ end of the primer are also in contact with the DNA polymerase, at residues R660, R587, R536, K508, and R487 (all positively charged amino acids), as well as E507 (a negatively charged amino acid). The substitution of the positively charged amino acids directly in contact with phosphate backbone of the primer in the closed conformation (R536, R587, or R660) has been reported to increase primer selectivity.^19^ However, when these mutations were combined to maximize selectivity, the polymerase activity of the resulting variants turned out to be compromised. This issue was resolved by introducing an additional mutation at E507, which interacts with the phosphate backbone of the sixth and seventh nucleotides of the primer’s 3′ ends. E507K amino acid substitution is known to stabilize the *Taq* polymerase–DNA binary complex, dramatically reduce the K_D_ by a factor of 90, improve polymerization responsiveness by a factor of approximately 1.7 during fast cycling PCR, and increase the resistance to PCR inhibitors.^27^^,^^28^ Accordingly, different combinations of E507, R536, and R660 were tested, and ultimately the E507K/R536K/R660V triple mutation was used for optimum AS amplification.

**Figure 6:**
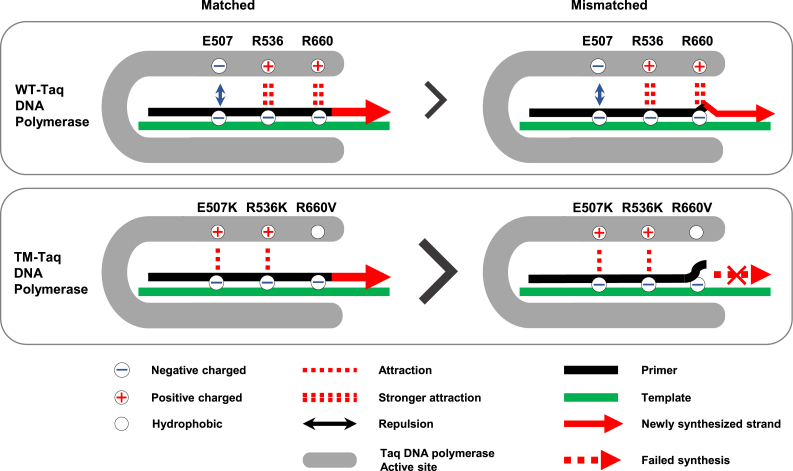
Triple-mutant (TM)-*Taq* DNA polymerase–based, allele discriminating priming system for higher mismatch discrimination. Simplified schematics showing the difference between wild-type (WT)- and TM-*Taq* DNA polymerase. WT-*Taq* enzyme extends even mismatched primer (although less efficiently), whereas TM-*Taq* enzyme extends only perfectly matched primer.

The improved performance by the TM-*Taq* DNA polymerase, therefore, is the combination of the reduced affinity of the enzyme to a mismatched primer–template complex achieved by R536K and R660V and the improved binding to a matched primer–template complex due to E507K mutation. The changes in these amino acids may hamper the primer extension, by releasing the mismatched primer from the active site, when the 3′ end of the primer has a single mismatch to the template, while successfully extending the matched primer (Figure 6). In contrast, the WT-*Taq* DNA polymerase still extends the primer even when the 3′ end of the primer has a mismatch, likely owing to the binding strength not being weak enough to release the mismatched primer from the template (Figure 6).

The results presented here are of particular significance when applied to early diagnosis of cancers. ctDNA refers to cell-free DNA fragments released from cancer cells, present in body fluids such as blood and cerebrospinal fluid. It has been used with increasing frequency to identify genomic alterations, to detect disease progression before clinical confirmation, and to monitor treatment response or resistance.^21^^,^^22^^,^^33^^,^^34^ The detectability of ctDNA is limited by tumor size, and thus sensitivity and specificity of the detection method are critical in early diagnosis when the tumor is small. Our TM-*Taq* DNA polymerase–based ADPS can improve ctDNA detection in a patient’s blood sample, as it provides high sensitivity of detecting cancer mutations even when they are present in only as low as 0.01% of 3 × 10^4^ genomic DNA copies (Figure 4C). Furthermore, its high selectivity/specificity for the matched primer–template complex against the mismatched reduces the potential for false-positive diagnosis. The application of our ADPS with TM-*Taq* DNA polymerase to clinical samples from patients with cancer has shown considerable promise and will be reported elsewhere. In addition, our system was also successfully applied to tissue-based genotyping for genetically engineered mice: *Pik3r2* knock-in^35^ and *Flag-Arx* knock-in mice generated with the CRISPR/Cas9 system (data not shown).^36^

A limitation of the ADPS system is that its performance is highly dependent on primer design, which can be solved by manipulating the length of the primer with a trial-and-error approach. Also, ADPS can be used only when the variant sequence of the template DNA is known, and thus it is not suitable for the discovery of emerging or novel cancer mutations during disease progression.

In summary, we have developed a TM-*Taq* DNA polymerase–based ADPS for ultra-rare genetic variant detection that is highly selective and sensitive. Our system can detect all types of 3′ end mismatch with a consistently higher sensitivity than the WT-*Taq* enzyme. This system may be useful for multiple applications such as SNP/single nucleotide variant genotyping, cancer diagnostics and monitoring, and rapid genotyping for genetically engineered cells or animals generated by CRISPR/Cas 9 genome editing.

## Supplemental Data

Supplemental Figure S1 Real-time quantitative PCR results for single nucleotide polymorphism detection by E507K/R536K and E507K/R660V mutant *Taq* DNA polymerases. Template: genomic DNA (50 ng per reaction) from buccal swab. Single nucleotide polymorphisms: *rs1015362* (CC genotype) (**A**) and *rs1408799* (TT genotype) (**B**). ΔC_T_: the difference of C_T_ (cycle threshold) values between mismatched (blue) versus matched primers (red). ΔRn, the difference of Rn (the fluorescence signal of the reporter probe normalized to that of the reference dye) values between the experimental versus the baseline signal.

Supplemental Figure S2 Real-time quantitative PCR results for cancer DNA mutation detection by R536K/R660V mutant *Taq* DNA polymerase. Templates: plasmid DNA harboring wild-type or mutant sequence of the *BRAF* and *JAK2* genes (1 × 10^6^ copies for *BRAF*, and 1 × 10^6^ copies for *JAK2* DNA) (*BRAF* V600E and *JAK2* V617F). Primers: each mutation-specific primer. ΔC_T_: the difference of C_T_ values between the wild-type (blue, mismatched) and mutant (red, matched) templates.

Supplemental Figure S3 Real-time quantitative PCR results for cancer DNA mutation detection by R536K, R536L, E507K/R536L/R660V, and E507K/R536K/R660V mutant *Taq* DNA polymerases. Templates: plasmid DNA harboring wild-type (*EGFR*) or mutant (*EGFR* L858R) sequence of the *EGFR* gene (3 × 10^4^ copies each). Primers: cancer mutant DNA-specific primer. ΔC_T_: the difference of C_T_ values between the wild-type (blue, mismatched) and mutant (red, matched) templates.

Supplemental Figure S4 Triple mutant (TM)-*Taq* DNA polymerase shows improved mismatch selectivity irrespective of mismatch types. Real-time quantitative PCR results of all three possible mismatch types of each primer containing T, G, or A at the 3′ end. Wild-type (WT) genomic DNA (genomic DNA) was used as a template and either WT-specific (red, match) or mutant-specific (blue, mismatch) primer was used in each reaction, by WT-, E507K-, or TM-*Taq* DNA polymerase. ΔC_T_: the difference of C_T_ (cycle threshold) values between mismatched (blue) versus matched primers (red). ΔRn: the difference of Rn (the fluorescence signal of the reporter probe normalized to that of the reference dye) values between the experimental versus the baseline signal.

Supplemental Figure S5 Optimization of triple mutant-*Taq* DNA polymerase (TM-*Taq* Pol) reaction. **A:** Primer length optimization: real-time quantitative PCR (qPCR) results comparing the performance of TM-*Taq* Pol with different lengths of primers. Template DNAs: plasmid DNAs (10,000 copies per reaction) harboring wild-type (WT) or each mutation sequence [*KRAS* G13D (c.38G>A), *PIK3CA* Q546L (c.1637A>T), and *PIK3CA* H1047R (c.3140A>G)] of the *KRAS* and *PIK3CA* genes. Primers: each mutation-specific primer with indicated lengths (the same 3′ end and different lengths at the 5′ direction). **B:** qPCR results comparing the performance of TM-*Taq* Pol with different concentrations of potassium chloride (KCl) and tetramethylammonium chloride (TMAC). Template DNAs: plasmid DNA (100,000 copies per reaction) harboring WT or mutation sequence [(*KRAS* Q61H (c.183A>C)] of the *KRAS* gene. **C:** qPCR results comparing the performance of TM-*Taq* Pol with different concentrations of ammonium sulfate [(NH_4_)_2_SO_4_]. The reaction buffer contained 50 mmol/L Tris-HCl (pH 8.8), 75 mmol/L KCl, 2.5 mmol/L magnesium chloride, 0.1% Tween 20 and 0.01% bovine serum albumin. Template: genomic DNA (50 ng per reaction) from buccal swab. Single nucleotide polymorphism: *rs4911414* (GG genotype). ΔC_T_: the difference of C_T_ values between the WT template (blue, mismatched) and the mutant template (red, matched).

Supplemental Figure S6 Schematic diagram illustrating triple mutant *Taq* DNA polymerase (TM-*Taq* DNA Pol)–based allele-discriminating priming system (ADPS) for cancer-related single nucleotide variant (SNV) detection. **A:** ADPS consists of three components: E507K/R536K/R660V TM-*Taq* DNA Pol, optimized primer–probe, and optimized buffer system. **B:** Only matched target sequence (eg, cancer mutation) is specifically amplified but not mismatched target (eg, wild type) by TM-*Taq* DNA Pol–mediated allele-specific PCR.

Supplemental Figure S7 Comparison of the detection sensitivity between the wild-type (WT)- and triple mutant (TM)-*Taq* DNA polymerase. Real-time quantitative PCR results by WT- and TM-*Taq* enzyme using the selected cancer mutant plasmid DNA templates mixed with the corresponding WT genomic DNA template at a different proportion [mutant (minor) allele fraction from 100% to 0%], and the mutant-specific primers. All three possible cases of the mismatch type for each base sequence (eg, T to C, G, and A) were evaluated by using the selected cancer genes indicated (eg, *KRAS* Q61R for T to C mismatch). ΔRn: the difference of Rn (the fluorescence signal of the reporter probe normalized to that of the reference dye) values between the experimental versus the baseline signal.
