# Supplementary figures and images for: Modified Taq DNA Polymerase for Allele-Specific Ultra-Sensitive Detection of Genetic Variants
Source: J Mol Diagn. 2022 Nov;24(11):1128–42. doi: 10.1016/j.jmoldx.2022.08.002 (PMC9746316; doi:10.1016/j.jmoldx.2022.08.002)

# Supplemental Figure S1

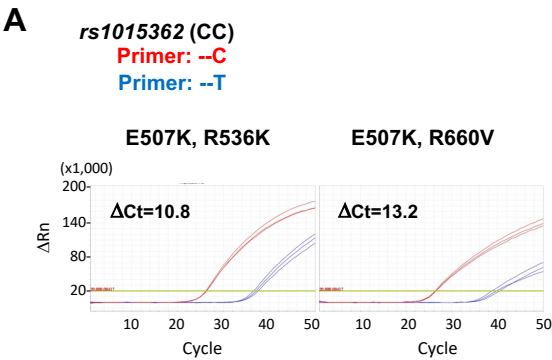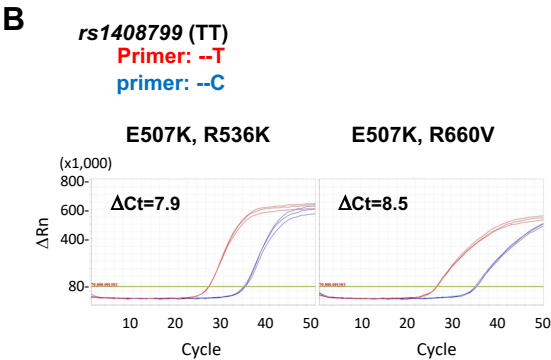

Supplement: Supplemental Figure S1 — Real-time quantitative PCR results for single nucleotide polymorphism detection by E507K/R536K and E507K/R660V mutant Taq DNA polymerases. Template: genomic DNA (50 ng per reaction) from buccal swab. Single nucleotide polymorphisms: rs1015362 (CC genotype) (A) and rs1408799 (TT genotype) (B). ΔCT: the difference of CT (cycle threshold) values between mismatched (blue) versus matched primers (red). ΔRn, the difference of Rn (the fluorescence signal of the reporter probe normalized to that of the reference dye) values between the experimental versus the baseline signal. [file mmc1.pdf]

# Supplemental Figure S2

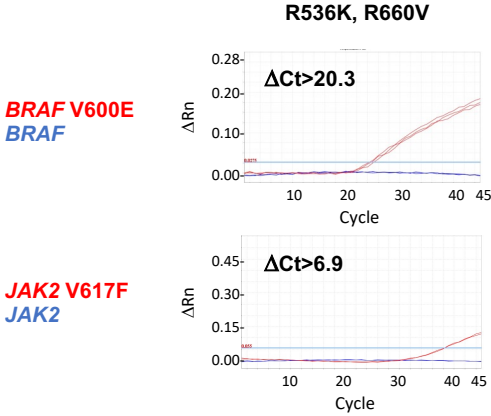

Supplement: Supplemental Figure S2 — Real-time quantitative PCR results for cancer DNA mutation detection by R536K/R660V mutant Taq DNA polymerase. Templates: plasmid DNA harboring wild-type or mutant sequence of the BRAF and JAK2 genes (1 × 10^6^ copies for BRAF, and 1 × 10^6^ copies for JAK2 DNA) (BRAF V600E and JAK2 V617F). Primers: each mutation-specific primer. ΔCT: the difference of CT values between the wild-type (blue, mismatched) and mutant (red, matched) templates. [file mmc2.pdf]

# Supplemental Figure S3

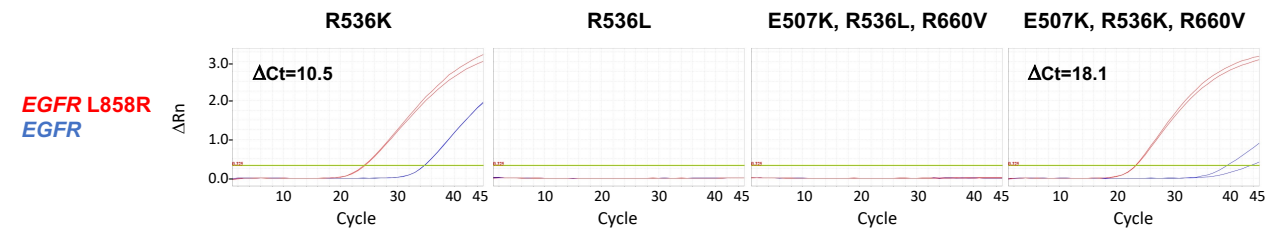

Supplement: Supplemental Figure S3 — Real-time quantitative PCR results for cancer DNA mutation detection by R536K, R536L, E507K/R536L/R660V, and E507K/R536K/R660V mutant Taq DNA polymerases. Templates: plasmid DNA harboring wild-type (EGFR) or mutant (EGFR L858R) sequence of the EGFR gene (3 × 10^4^ copies each). Primers: cancer mutant DNA-specific primer. ΔCT: the difference of CT values between the wild-type (blue, mismatched) and mutant (red, matched) templates. [file mmc3.pdf]

# Supplemental Figure S6

A

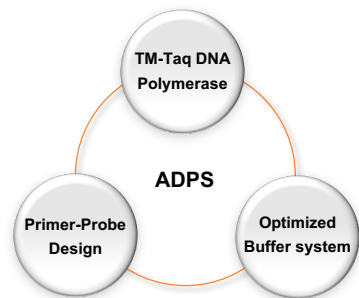

B

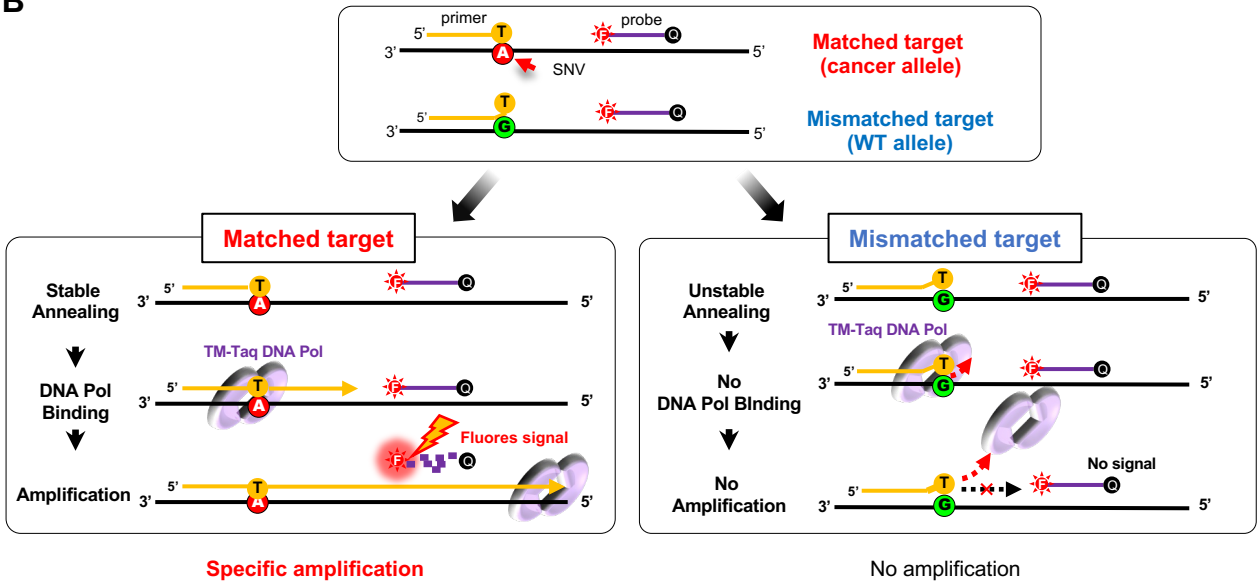

Supplement: Supplemental Figure S6 — Schematic diagram illustrating triple mutant Taq DNA polymerase (TM-Taq DNA Pol)–based allele-discriminating priming system (ADPS) for cancer-related single nucleotide variant (SNV) detection. A: ADPS consists of three components: E507K/R536K/R660V TM-Taq DNA Pol, optimized primer–probe, and optimized buffer system. B: Only matched target sequence (eg, cancer mutation) is specifically amplified but not mismatched target (eg, wild type) by TM-Taq DNA Pol–mediated allele-specific PCR. [file mmc6.pdf]

# Supplemental Figure S7

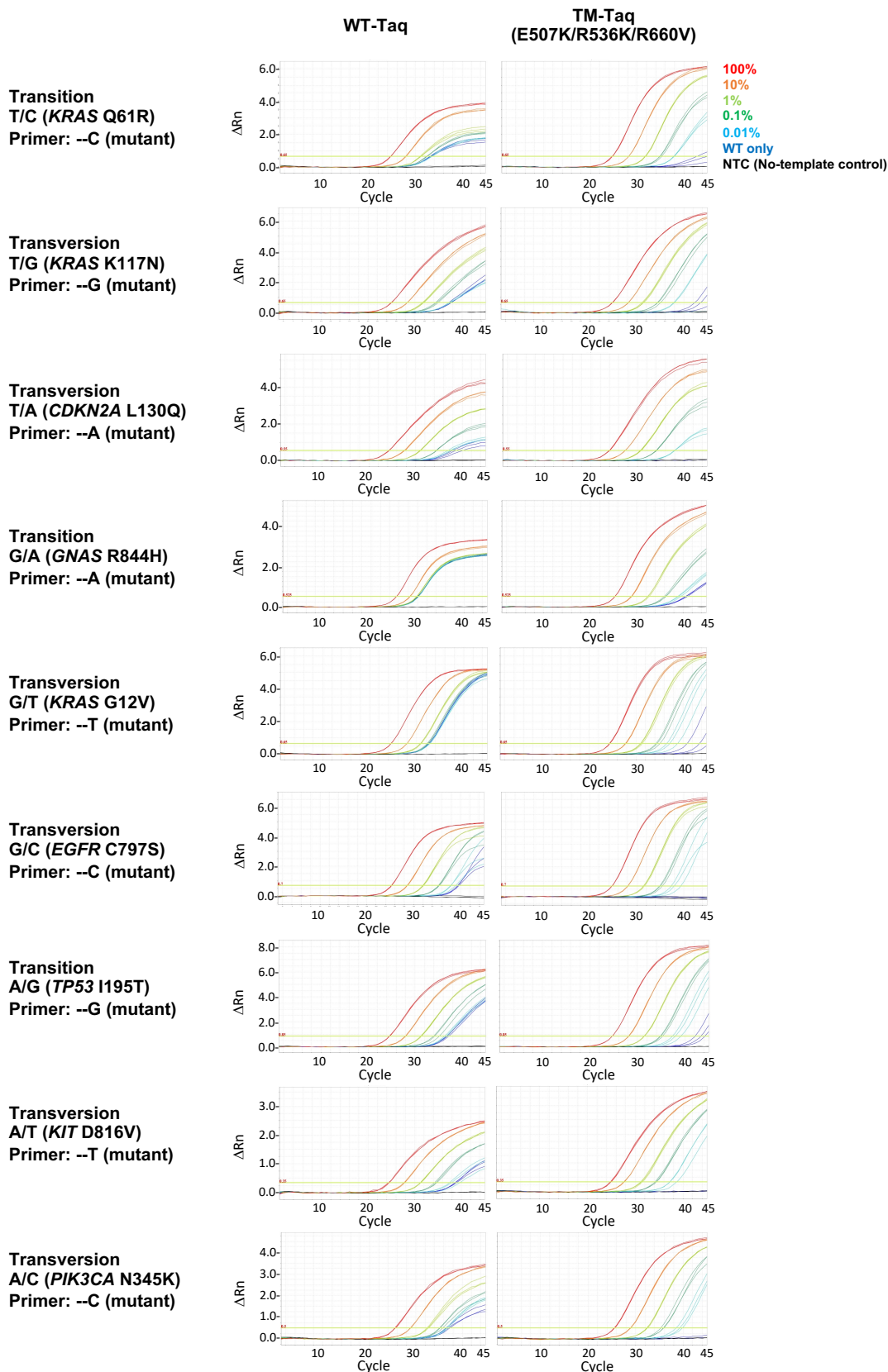

Supplement: Supplemental Figure S7 — Comparison of the detection sensitivity between the wild-type (WT)- and triple mutant (TM)-Taq DNA polymerase. Real-time quantitative PCR results by WT- and TM-Taq enzyme using the selected cancer mutant plasmid DNA templates mixed with the corresponding WT genomic DNA template at a different proportion [mutant (minor) allele fraction from 100% to 0%], and the mutant-specific primers. All three possible cases of the mismatch type for each base sequence (eg, T to C, G, and A) were evaluated by using the selected cancer genes indicated (eg, KRAS Q61R for T to C mismatch). ΔRn: the difference of Rn (the fluorescence signal of the reporter probe normalized to that of the reference dye) values between the experimental versus the baseline signal. [file mmc7.pdf]
