# Supplementary material for: Modified Taq DNA Polymerase for Allele-Specific Ultra-Sensitive Detection of Genetic Variants
Source: J Mol Diagn. 2022 Nov;24(11):1128–42. doi: 10.1016/j.jmoldx.2022.08.002 (PMC9746316; doi:10.1016/j.jmoldx.2022.08.002)

Supplemental Figure S4

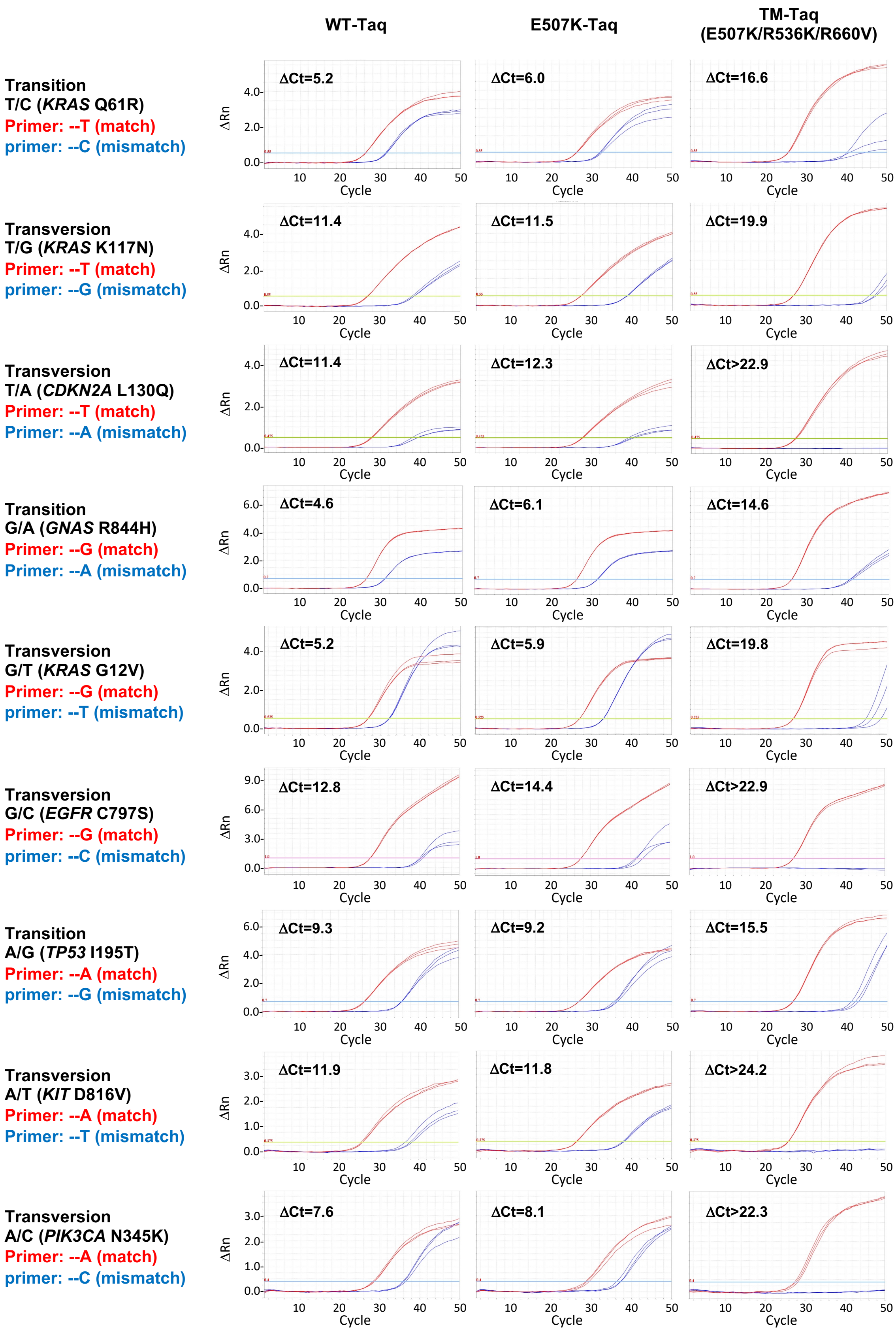

Supplement: Supplemental Figure S4 — Triple mutant (TM)-Taq DNA polymerase shows improved mismatch selectivity irrespective of mismatch types. Real-time quantitative PCR results of all three possible mismatch types of each primer containing T, G, or A at the 3′ end. Wild-type (WT) genomic DNA (genomic DNA) was used as a template and either WT-specific (red, match) or mutant-specific (blue, mismatch) primer was used in each reaction, by WT-, E507K-, or TM-Taq DNA polymerase. ΔCT: the difference of CT (cycle threshold) values between mismatched (blue) versus matched primers (red). ΔRn: the difference of Rn (the fluorescence signal of the reporter probe normalized to that of the reference dye) values between the experimental versus the baseline signal. [file mmc4.pdf]
