# Supplementary material for: Modified Taq DNA Polymerase for Allele-Specific Ultra-Sensitive Detection of Genetic Variants
Source: J Mol Diagn. 2022 Nov;24(11):1128–42. doi: 10.1016/j.jmoldx.2022.08.002 (PMC9746316; doi:10.1016/j.jmoldx.2022.08.002)

Supplemental Figure S5

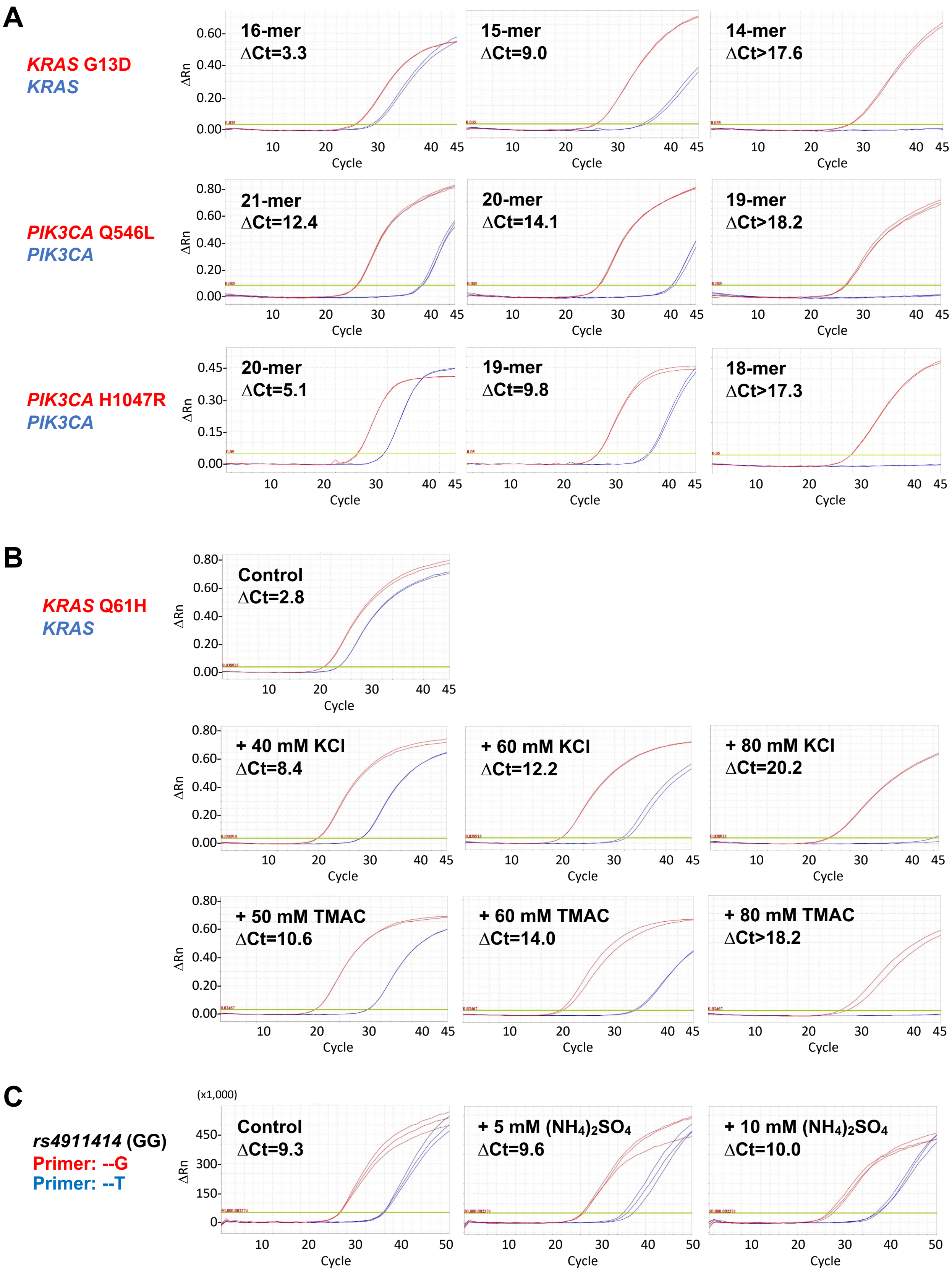

Supplement: Supplemental Figure S5 — Optimization of triple mutant-Taq DNA polymerase (TM-Taq Pol) reaction. A: Primer length optimization: real-time quantitative PCR (qPCR) results comparing the performance of TM-Taq Pol with different lengths of primers. Template DNAs: plasmid DNAs (10,000 copies per reaction) harboring wild-type (WT) or each mutation sequence [KRAS G13D (c.38G>A), PIK3CA Q546L (c.1637A>T), and PIK3CA H1047R (c.3140A>G)] of the KRAS and PIK3CA genes. Primers: each mutation-specific primer with indicated lengths (the same 3′ end and different lengths at the 5′ direction). B: qPCR results comparing the performance of TM-Taq Pol with different concentrations of potassium chloride (KCl) and tetramethylammonium chloride (TMAC). Template DNAs: plasmid DNA (100,000 copies per reaction) harboring WT or mutation sequence [(KRAS Q61H (c.183A>C)] of the KRAS gene. C: qPCR results comparing the performance of TM-Taq Pol with different concentrations of ammonium sulfate [(NH4)2SO4]. The reaction buffer contained 50 mmol/L Tris-HCl (pH 8.8), 75 mmol/L KCl, 2.5 mmol/L magnesium chloride, 0.1% Tween 20 and 0.01% bovine serum albumin. Template: genomic DNA (50 ng per reaction) from buccal swab. Single nucleotide polymorphism: rs4911414 (GG genotype). ΔCT: the difference of CT values between the WT template (blue, mismatched) and the mutant template (red, matched). [file mmc5.pdf]
