# Supplementary material for: Modified Taq DNA Polymerase for Allele-Specific Ultra-Sensitive Detection of Genetic Variants
Source: J Mol Diagn. 2022 Nov;24(11):1128–42. doi: 10.1016/j.jmoldx.2022.08.002 (PMC9746316; doi:10.1016/j.jmoldx.2022.08.002)
Supplement: Supplemental Table S1 [file mmc8.docx]

**Supplemental Table S1**. *Taq* DNA polymerase variants used in this study

| Subset | Target | E507K | R536K | R536L | E507K/  R536K | E507K/  R660V | R536K/  R660V | E507K/R536L/  R660V | E507K/R536K/  R660V |
| --- | --- | --- | --- | --- | --- | --- | --- | --- | --- |
| SNP genotyping | *rs1015362* | ✓ |  |  | ✓ | ✓ |  |  | ✓ |
|  | *rs1408799* | ✓ |  |  | ✓ | ✓ |  |  | ✓ |
|  | *rs3918242* | ✓ |  |  |  |  | ✓ |  | ✓ |
| Cancer  Mutation  detection | *BRAF* V600E | ✓ |  |  |  |  | ✓ |  | ✓ |
|  | *JAK2* V617F | ✓ |  |  |  |  | ✓ |  | ✓ |
|  | *EGFR* L858R | ✓ | ✓ | ✓ |  |  |  | ✓ | ✓ |
|  | *EGFR* Ex19Del | ✓ |  |  |  |  |  |  | ✓ |
|  | *EGFR* Ex20Ins | ✓ |  |  |  |  |  |  | ✓ |
|  | *KRAS* Q61H |  | ✓ | ✓ |  |  |  | ✓ | ✓ |
|  | *KRAS* G13D |  |  |  |  |  |  |  | ✓ |
|  | *PIK3CA* Q546L |  |  |  |  |  |  |  | ✓ |
|  | *PIK3CA* H1047R |  |  |  |  |  |  |  | ✓ |

✓, used in this study
